# Supplementary material for: A scoping review protocol on in vivo human plastic exposure and health impacts
Source: Syst Rev. 2022 Jul 5;11:137. doi: 10.1186/s13643-022-02010-6 (PMC9258212; doi:10.1186/s13643-022-02010-6)
Supplement: Supplementary file 2 — Additional file 2. Draft data extraction forms. [file 13643_2022_2010_MOESM2_ESM.pdf]

Additional File II:

DRAFT DATA EXTRACTION FORM

1. Year of publication
2. Country of publication for first and last authors
3. Investigated population(s)
    a. Country/countries
    b. Individuals/pairs/families?
    c. Gender
    d. Age
    e. General/special risk (in terms of exposure)
4. Plastic exposures
5. Health outcome measures
6. Study design
7. Notes
